# Tensile Mechanical Behaviour of Multi-Polymer Sandwich Structures via Fused Deposition Modelling

**DOI:** 10.3390/polym12030651

**Published:** 2020-03-12

**Authors:** David Moises Baca Lopez, Rafiq Ahmad

**Affiliations:** Laboratory of Intelligent Manufacturing, Design, and Automation (LIMDA), Department of Mechanical Engineering, University of Alberta, 9211 116 St NW, Edmonton, AB T6G 1H9, Canada; bacalope@ualberta.ca

**Keywords:** additive manufacturing, fused deposition modelling, multi-material 3D printing, sandwich structure

## Abstract

The application of single homogeneous materials produced through the fused deposition modelling (FDM) technology restricts the production of high-level multi-material components. The fabrication of a sandwich-structured specimen with different material combinations using conventional thermoplastics such as poly (lactic acid) (PLA), acrylonitrile butadiene styrene (ABS) and high impact polystyrene (HIPS) through the filament-based extrusion process can demonstrate an improvement on its properties. This paper aims to assess among these materials, the best material sandwich-structured arrangement design, to enhance the mechanical properties of a part and to compare the results with the homogeneous materials selected. The samples were subjected to tensile testing to identify the tensile strength, elongation at break and Young’s modulus of each material combination. The experimental results demonstrate that applying the PLA-ABS-PLA sandwich arrangement leads to the best mechanical properties between these materials. This study enables users to consider sandwich structure designs as an alternative to manufacturing multi-material components using conventional and low-cost materials. Future work will consider the flexural tests to identify the maximum stresses and bending forces under pressure.

## 1. Introduction

Additive manufacturing (AM) began in 1987 when it was known as generative manufacturing or rapid prototyping [1]. Since then, it has represented a robust manufacturing development and process of design for products in a wide variety of sectors, including aerospace, biomedical and manufacturing [2,3,4,5]. AM technology can fabricate accurately and strengthened components in a fast production, being able to either be combined with traditional manufacturing techniques known as hybrid technologies, i.e., subtractive and additive remanufacturing [6,7], or even as an option to displace them shortly [8]. Presently, extensive research on fused deposition modelling (FDM) materials is being carried out on thermoplastic polymers and composites due to their cost-effective, lightweight, and high strength-to-weight ratio [9]. Material selection methodologies have been developed to choose optimal materials at the early design stages of the AM processes [10]. Berto et al. [11] conducted a design tool for polymers in particular polyetheretherketone (PEEK) specimens with different notch geometries to assess the tensile and fatigue behaviour on load-bearing applications. Marsavina et al. [12] studied the tensile properties of two different polyamide materials (PA2200 and Alumide) based on the AM process of selective laser sintering (SLS). The authors established the same technological parameters except for the building orientation envelope of the sample. Moreover, research is being done by combining materials, including polylactic acid and polycarbonate (PLA-PC), acrylonitrile butadiene styrene and polycarbonate (ABS-PC) and polyethylene and polypropylene (PE-PP) [13].

In recent years, the fabrication of multi-material polymer-based products and sandwich structure methods applied to the FDM process have brought the attention of the industry and research community. The benefits of multi-material enable the performance of smart components by improving 3D printing quality through topology optimization of different process parameters [14,15]. Thus, producing multi-material components may enhance the mechanical properties, enable new functionality and improve the performance of the AM process. [16]. In addition, sandwich structures made of different polymers combinations have been considered an excellent option to achieve various material properties for customized products [17], e.g. lightweight interior components in the automotive sector. Usually, sandwich structures are applied to composite materials, which consist of outer skins (thin facings) made of high-strength material sandwiching the inner core made of a lightweight material [18]. The inner core usually consists of a honeycomb configuration due to its weight efficiency. However, it can encounter problems such as water intrusion and delamination [19]. Daniel and Abot [20] suggested that varying the materials for the skin and core can enable the desired stiffness and strength. Herranen et al. [21] concluded that the optimal design to develop a lightweight sandwich composite appears to be more accurate in the core material than in the core layer thickness. For homogenous thermoplastics on the FDM process, Lanzotti et al. and Chacón et al. [22,23] studied the influence of the process parameters (layer thickness, flow rate, deposition speed, feed rate and build orientation) for single specimens made of PLA. In the first paper, it was concluded that all the fibers had to be oriented along the loading line to maximize the value of Young’s modulus and stiffness. In the second paper, it was realized that on-edge samples (build orientation) showed the best performance in terms of strength, stiffness and ductility. Besides, as the layer thickness and feed rate are increased, ductility is decreased. Kuznetsov et al. [24] studied the influence of shell, base thickness and infill density of PLA to optimize the geometry of an FDM 3D printed part that can withstand higher loads. It was found that, optimizing these process parameters and adding certain volume features such as fillets, roundness and smooth contours can increase the part strength more than double of the original force that was required to fracture the same part and with a significant reduction of part mass as well. Wang et al. [25] conducted a micromechanical model with an experimental investigation to examine the correlation between the influence of internal density pores and the influence of the process parameters (raster angle and extrusion width) on the mechanical properties of PLA produced by the FDM process. The results demonstrate that the model can help future designers predict the elastic properties of a produced part without wasting material in future destructive testing.

Fernandez-Vicente et al. [26] evaluated the strength of different mesostructures for ABS manufactured with the FDM process. For the evaluation, a tensile test was conducted with various parameters including the material densities and infill patterns to select the optimal specimen. The best combination to obtain the highest tensile strength was the rectilinear pattern with a 100% infill density. Heechang [27] discussed the use of single and dual materials for the development of multi-material printing. The paper proposed the influence of equipment variables, material ratio percentage and different structural arrangements to identify and improve the mechanical behaviour of the specimens. Lopes et al. [28] examined the effect of the boundary interface formed in different zones using dual nozzles to fabricate multi-material parts. The tested specimens showed a decrease in their tensile strength and Young’s modulus due mainly to switching between extruders. The author suggested that a proper design for the boundary interface must be implemented to achieve higher mechanical properties. Singh et al. and Kumar et al. [29,30] conducted mechanical tests to find the best material combination among ABS, PLA and HIPS. The three materials were printed in the same geometry as a stack of different multi-layers, and the twin extrusion method (TSE) was implemented. The conducted study was influenced by the infill percentage and printing speed of each material combination. Saad [31] fabricated a sandwich structure with ABS and PLA to test the variation of their mechanical and physical properties. The study consisted of different infill percentages of honeycomb cores to identify the effect of pore size volume on the mechanical properties and to validate the weight benefit. The results of the tensile and bending test showed that the tensile strength increases as long as the infill density increases while the stiffness remains constant with bending stress. Brischetto et al. [32] proposed three- and four-point bending tests for ABS and PLA using sandwich structures. The proposed experimental analysis was influenced by the infill patterns (honeycomb and homogenous) as core layers and by the number of extruders (two) to fabricate the specimens. All these parameters had a significant influence on their modulus of elasticity especially in those specimens made of ABS skins and PLA honeycomb core. Santosh et al. [33] prepared multilayers structures using ABS with PLA to improve their mechanical behaviour. The mechanical properties were evaluated using the tensile test, compressive test, bending test, microhardness and surface roughness. De Souza et al. [34] studied the mechanical properties of blending ABS and HIPS to analyze the effect of shot size and particle size via injection molding. The results suggest that increasing ABS in the ABS/HIPS blend leads to an increase in the tensile strength and Young’s modulus, but it decreases the elongation at break. Dinesh et al. [35] investigated the mechanical properties of a sandwich structured using carbon fibers reinforced composites with different combs such as aluminum honeycomb, Rohacell and high density polyurethane (HDPU) foam core. The tensile results observed that the aluminum honeycomb core could easily tear in axial loading compared to the rest; however, for the bending test, it presented the highest bending load among the other materials. Galatas et al. [36] proposed to enhance the mechanical properties of composite sandwich structures for ABS with carbon fiber reinforced polymer (CFRP). The parameters considered in the study were the infill densities and the number of CFRP layers. The ultimate strength of these composite dog-bone specimens was evaluated, employing a tensile test. The experimental results were compared with a developed artificial intelligence neural network, and the influence of other properties such as Young’s modulus and specific strength was also discussed. Mazzanti et al. [37] analyzed an extensive review on the mechanical properties of 3D printing polymers containing natural fillers. They found that classical thermoplastics filaments such as PLA and ABS perform negatively when filler content is applied and therefore a reduction on the mechanical properties is presented, while uncommon FDM plastics present better performance. Caminero et al. [38] conducted a study to evaluate the effect of nanoparticle reinforcements on the mechanical properties of polymeric materials, especially for PLA-graphene. The build orientation of the printed specimen was critical to obtain the best mechanical performance. The PLA-graphene composite showed the best mechanical values in terms of tensile and flexural stress and the study indicated that printing reinforced composite materials concerning conventional manufacturing processes can increase the mechanical properties of a part.

In the authors’ previous work, tensile tests for single material specimens made of PLA [22,23] and ABS [26] were conducted. The proposed methodologies using image and statistical analyses were used to evaluate the mechanical properties (tensile strength, tensile strain and elastic modulus) of the produced samples following the ASTM 638 type. Besides, most of the experimental tests employed for the sandwich structures in the past were conducted by considering the ASTM D790 and the ASTM D6272 standards for flexural properties of un-reinforced and reinforced thermoplastics, respectively. Based on the literature review and to the best of the authors’ knowledge, to date, the use of sandwich structure applied to different combinations of materials via FDM for improving the mechanical properties is lacking, which is the aim of this paper.

The present paper investigates and tests the influence of sandwich structures applied to conventional 3D printing materials to improve the mechanical properties of 3D printed prototypes. The new sandwich structure here proposed are specimens embedding rectilinear cores pattern with a 100% infill density and applying multiple independent nozzle extruders for each material within the same carriage. This new scheme could provide a piece of knowledge for comparison on the effect of combining conventional materials (ABS, PLA and HIPS) as a sandwich structure to achieve and enhance higher strength of polymeric parts, which can be used for various applications, e.g. unmanned aerial vehicles (UAV). The tensile experiments aimed to identify whether the use of conventional materials printed as a sandwich structure is suitable and beneficial to implement and able to improve their mechanical behaviour (tensile strength, elongation at break and Young’s modulus) when compared with a single material 3D printed part. Therefore, the contribution of this paper lies in finding the best final product performance of the selected materials.

## 2. Materials and Methods

**Figure 1:**
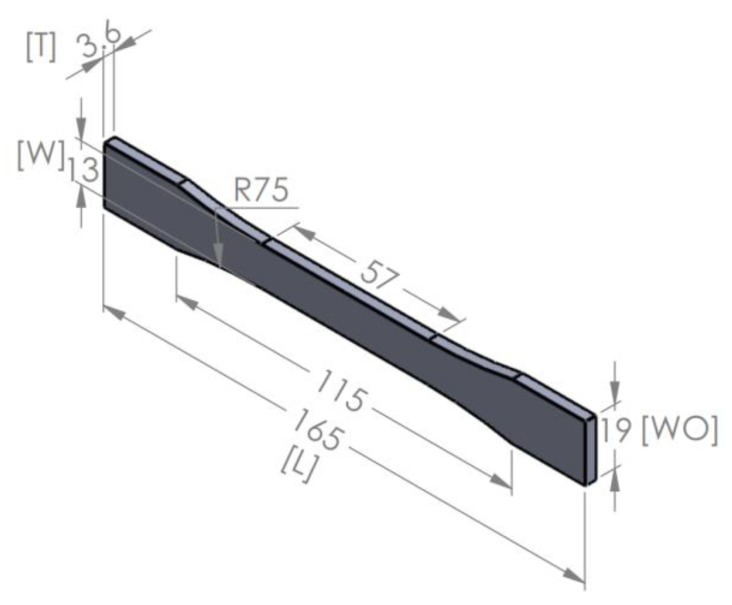
Standard test specimen in mm.

The first material selected for this study was PLA. It is widely used in FDM 3D printing [39] and provides some benefits against other polymers such as environmentally friendly material, sustainable, biocompatible and excellent plasticity in long-term use [40]. The second material implemented was ABS. For the extrusion process, ABS provides excellent heat and high resistance, low thermal conductivity, toughness and compatibility with other materials [41]. Lastly, high impact polystyrene (HIPS) presents high impact strength and low tensile strength and provides simplicity for its fabrication and machinability [29]. For this study, it was aimed to assess if these materials could enhance the strength of a part by investigating the effect of different sandwich arrangements in the same geometry. Understanding the behaviour of conventional materials by heat fusion as a single batch production will enable in the future the use of more complex/engineering materials for FDM 3D printing composites. The method proposes a sandwich structure with a total thickness of 3.6 mm. The two outer skins have a global thickness of 2.4 mm and the inner core a thickness of 1.2 mm made of a different polymer with a rectilinear infill pattern and 100% density. The materials used to manufacture the test specimens were spools of 1.75 mm diameter of ABS, PLA and HIPS. The mechanical properties of these materials are reported in Table 1 [22,42,43]. The filaments were all obtained through commercial suppliers. The specimens were fabricated following the D638-Type 1 standard [44] to analyze how the structural arrangement determines a change in their tensile strength. The main dimensions of the specimen are shown in Figure 1.

Firstly, specimens were printed as a single homogeneous material with fixed parameters to be compared with the multi-material specimens. Secondly, the combination of materials for the sandwich structure was performed with four-layer sections of each material. It contained symmetric 1.2 mm thick polymeric inner and outer cores (four layers of 0.3 mm thick per material) giving a total dimension of a 3.6 mm thick specimen in accordance with the dimensions of the standard test method for tensile properties of plastics (ASTM D638-Type 1 standard) [44]. All specimens were printed in a flat direction on the XY plane with a rectilinear pattern, stacking sequence of (45°/−45°) and vertically upwards layer by layer within the Z direction.

**Figure 2:**
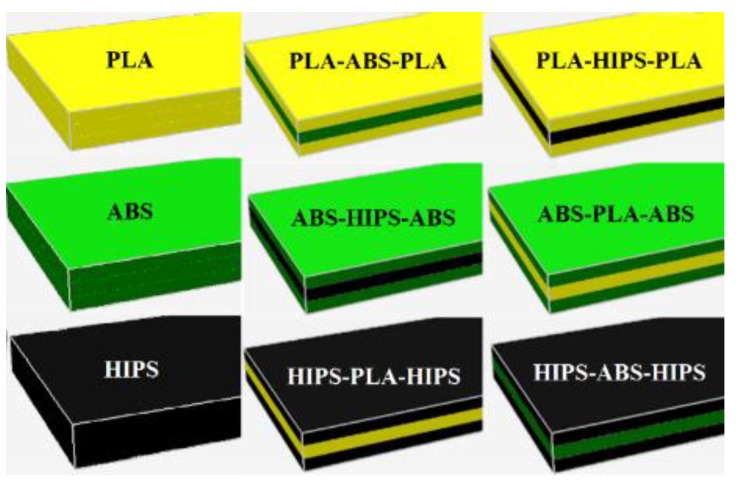
Geometry for a typical specimen as a single material and as a sandwich structure part (three sections with equal thickness).

The material configurations to fabricate the sandwich structure specimen are shown below. Figure 2 illustrates the specimen designs for homogeneous materials and sandwich structure material combinations. In addition, the printed parameters carried out to print the specimens are summarized in Table 2 [22]. The values of the most printing parameters were based on other experimental studies [22,26,45]. The parameters were carefully selected to be optimal for all printed specimens and by following the recommended printing specifications from the 3D printer manufacturers. For the bed temperature, for the PLA printed on a glass surface, a moderate adhesion force range based on previous studies is between 80 and 120 °C [46]. Other studies recommend that, for the ABS, the bed temperature should be set approximately 90–93 °C to minimize the warpage while printing [47]; therefore, a bed temperature of 90 °C for all materials was selected. Adding more parameters increases the complexity of the test, therefore for this particular study the air gap was established as zero (beads just touch).

- PLA-ABS-PLA and PLA-HIPS-PLA
- ABS-PLA-ABS and ABS-HIPS-ABS
- HIPS-PLA-HIPS and HIPS-ABS-HIPS

## 3. Results

### Experimental

**Figure 3:**
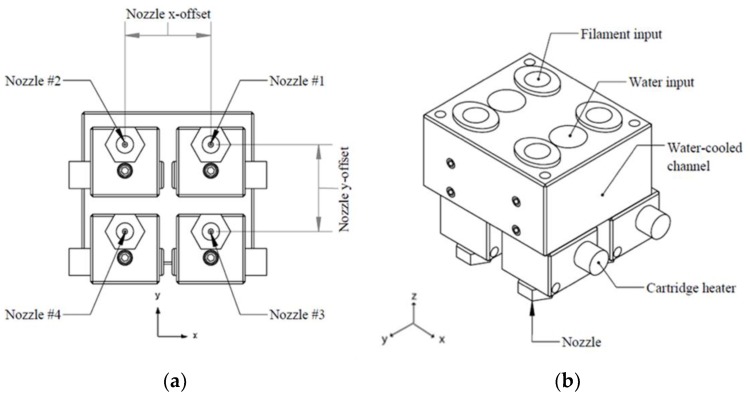
Illustration of the multi-nozzle module: (**a**) bottom view X-Y offsets; and (**b**) isometric view.

**Figure 4:**
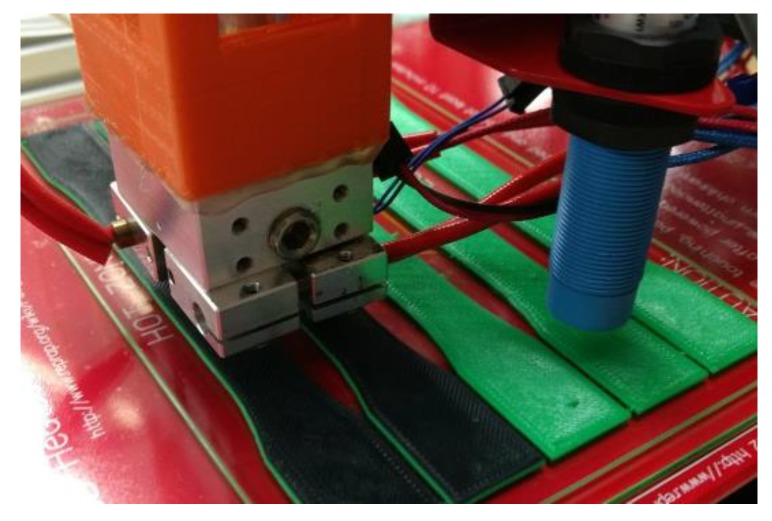
Multi-nozzle extruder while printing ABS-HIPS-ABS specimen.

For the present study, a multi-material 3D printer was developed and customized to allow the use of multiple filaments. It was necessary to integrate a multi-nozzle head extruder to enable producing different materials. The extrusion unit consisted of four nozzles within the same carriage. The module is based on the Bowden extrusion system [48], where the extruder mechanism is away from the printer’s heated head. The hot end of the extrusion nozzle integrated a water-cooled channel that serves as a heatsink to keep it continuously cooled while printing. The nozzles used on this module had a die diameter of 0.4 mm each. It was necessary to find the exact distance (height and width) and offset values between each nozzle to ensure that, once it deposits material, an overlap between layers exists (see Figure 3). To avoid nozzle influence while printing, the system disables the idle nozzle and controls the pressure to avoid material oozing (retracts the filament) and prevent contamination. In addition, nozzle leveling was carried out using a capacitive sensor, which was enabled in the Z-axis. Figure 4 shows the multi-nozzle extruder with the sensor and the specimens while printing.

The printed parts were measured before tested using an advanced Mitutoyo caliper. The measurements were compared with the exact dimensions of the CAD model design (Figure 1). All specimens were printed in a flat direction on the XY plane with a rectilinear pattern, stacking sequence of (45°/−45°) and vertically upwards layer by layer within the Z direction. Table 3 shows the average value of the dimensions from the manufactured test specimens, which were measured twice to validate the results for further analysis. Based on the obtained values between the homogeneous and different material combinations, no significant variations were observed. Hence, the results provide high precision and align with material fabrication.

The machine used to perform the tensile tests was the Instron 5966. It was configured with a minimum speed of 2 mm/min, a load cell of 10 kN and a gripper with a maximum load of 5 kN. The selection of the speed rate was in accordance with the loading rate used in other studies [22,23,25,26,49,50].

**Figure 5:**
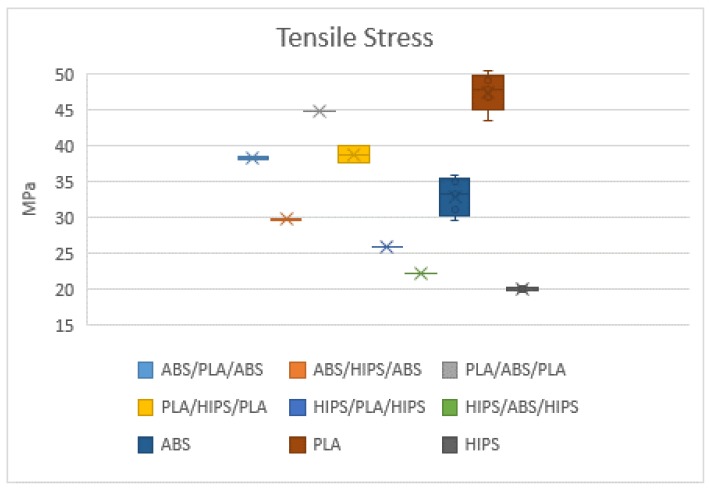
Mean tensile stress of all specimen tested.

**Figure 6:**
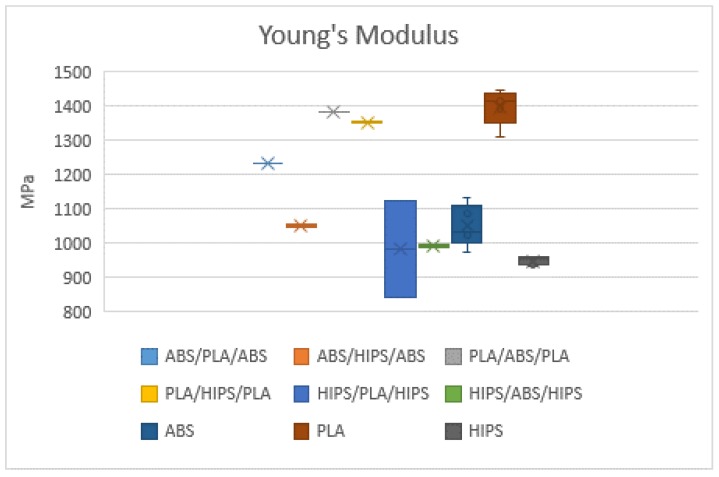
Mean Young’s modulus of all specimen tested.

**Figure 7:**
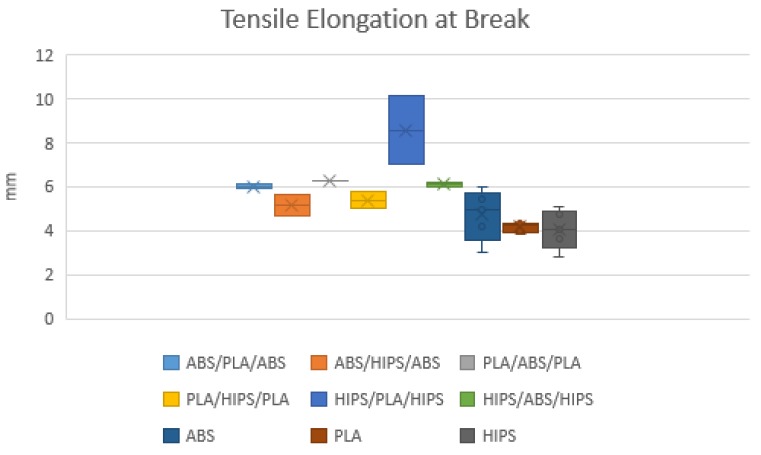
Mean tensile elongation at break of all specimen tested.

The specimens were loaded along the longitudinal axis until failure. According to the standard and for the evaluation of dispersion, five specimens were printed for each material configuration. In, total, 45 specimens were printed to validate the results. The mechanical properties considered for the comparison were the tensile strength, Young’s modulus and the elongation, as shown in Figure 5, Figure 6 and Figure 7, respectively.

The mechanical results obtained from the tensile test show differences between the homogeneous materials and the different combinations of sandwich structures. PLA demonstrated the highest tensile strength (47.46 MPa) and Young’s modulus (1396.90 MPa) among all specimens but presented the lowest elongation at break (4.16 mm). HIPS exhibited plastic deformation and showed the lowest tensile strength (20.06 MPa) and Young’s modulus (933.33 MPa) but had a higher elongation at break (6.69 mm) when compared to the other two. Moreover, it was expected that the performance of combining different materials would have a positive impact on the mechanical properties in comparison to the single material ones. In general, these three types of specimens showed higher ultimate strength and elastic modulus when fabricated with the sandwich configuration compared to a single material. Among these specimens, the combination of PLA-ABS-PLA had the best tensile strength and Young’s modulus with means of 44.40 and 1364.57 MPa, respectively. This behaviour may be due that both polymers are considered rigid materials. Besides, this combination had a significant improvement in the elongation at break with a mean of 6.14 mm. Considering that PLA had the highest tensile strength but the lowest elongation followed by ABS as homogeneous materials, it was observed that combining these two materials as a sandwich structure where PLA was printed as the outer skins and ABS as the inner core of the specimen provided a significant performance combining their properties. Secondly, PLA-HIPS-PLA had slightly higher tensile strength and Young’s modulus (38.77 and 1351.27 MPa) compared to ABS-PLA-ABS with a tensile strength of 38.28 MPa and Young’s modulus of 1232.96 MPa. In addition, the highest elongation at break exhibited from these three specimens was 6.14 mm from the PLA-HIPS-PLA sandwich structure.

## 4. Discussion

**Figure 8:**
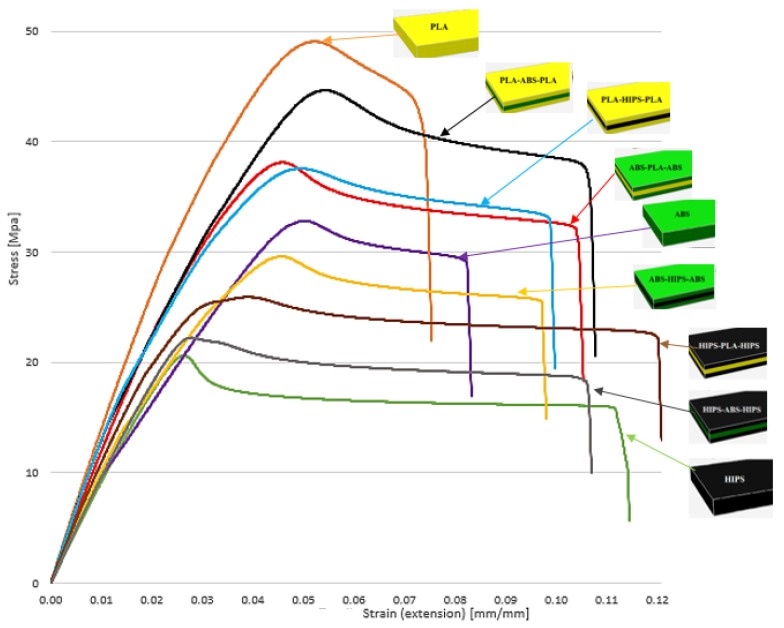
Stres–strain curve diagram of all the specimens tested.

Figure 8 compares the stress–strain curves of all the specimens. The analysis shows that the specimens at the beginning of the test presented a linear trend surpassing the stress of 15–20 MPa. Consequently, this trend changed considerably in different stages for each specimen when the ultimate strength was reached. The specimens with HIPS skins and ABS rectilinear core presented the worst performance with a mean tensile stress of 22.21 MPa and Young’s modulus of 992.02 MPa, which is less than 50% and 28%, respectively, of the best sandwich structure tested, which was PLA-ABS-PLA. Similar behaviour was encountered with the specimens with HIPS skins and PLA core. They showed a low mean tensile strength of 25.87 MPa and Young’s modulus of 981.45 MPa but performed better when reaching the highest elongation of all with a mean of 8.57 mm.

**Figure 9:**
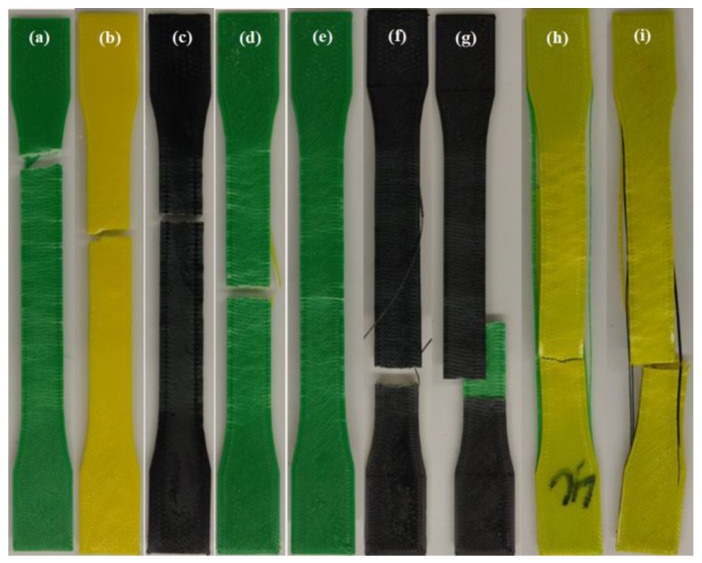
Examples of failure tested specimens: (**a**) ABS; (**b**) PLA; (**c**) HIPS; (**d**) ABS-PLA-ABS; (**e**) ABS-HIPS-ABS; (**f**) HIPS-PLA-HIPS; (**g**) HIPS-ABS-HIPS; (**h**) PLA-ABS-PLA; and (**i**) PLA-HIPS-PLA.

Figure 9 show representative examples of the tested specimens illustrating the failure mode. The fracture in all specimens shows the different stress distributions of the material combinations. It can be seen by comparing the specimens in Figure 9d,g,h that the fracture reveals the cracking pattern generated closely to failure.

The infill fibers of the skin layers deform rapidly and absorb the stress, causing to break the bonds within materials. The location of the ruptures of the sandwich-structured specimens was mostly generated near the jaws of the test equipment, and elsewhere for two cases (ABS-PLA-ABS and ABS-HIPS-ABS). In addition, it was determined that the weakest sections of the specimens in which the fractures occurred were due to the boundary interface within the transition between different regions of the specimen. In addition, the fractures occurred due to the boundary interface between materials. Despite this condition, using a sandwich structure arrangement for conventional materials can improve the mechanical properties, as demonstrated and described previously. The generated matrix of the corresponding results of the different sandwich structures is shown in Table 4 The data listed in the table confirm the results obtained from the box plot graphs and the stress–strain diagram. It is considered that the effect of interfacial energy is fundamental to identify the influence of layer bonding between different thermoplastic materials. In addition, the importance of forensic analysis to determine the cause of failure such as delamination is also critical. Therefore, the authors will investigate the analysis of interfacial energy with an experimental study in future work.

## 5. Conclusions

This research presented a unique polymer-based sandwich structure to evaluate the tensile properties of different material combinations produced via the FDM process. The aim of the study was applied to the fabrication of rectilinear infill cores and outer skins of several materials via multiple nozzle extruders. Experimental findings in the 3D printing process showed that the best sandwich-structured arrangement was the combination of outer skins of PLA and ABS cores. The average values were 44.40 MPa for the tensile strength and 1364.25 MPa for Young’s modulus. In addition, the elongation at break (6.14 mm) for this configuration was higher when compared to a homogeneous material. Specimens with outer skins of HIPS and ABS cores showed the lowest performance compared to the rest sandwich specimens. The mechanical behaviour between the sandwich structures is different. The change of the material arrangement determines mainly the tensile strength, elongation at break and stiffness between 30% and 50%.

Ultimately, this study demonstrated the capabilities and flexibilities of conventional 3D printing materials to be used to improve the efficiency of an AM product. Therefore, the use of the sandwich structure applied to conventional 3D printing materials gain applicability in generating functional parts that can withstand tensile loading for longer periods over single homogenous materials by integrating the properties of two different materials in the same part. It can also provide higher elongation at break, which can absorb energy and therefore give long-term use before failing. The advantages of multi-material and sandwich structures methods are suitable to implement and able to achieve the requirements of various applications using low-cost materials.

## Figures and Tables

**Table 1:** Typical properties of ABS, PLA and HIPS.

Material	PLA	ABS	HIPS
**Composition**	Polylactic acid	Acrylonitrile butadiene styrene	High-impact polystyrene
**Physical properties**		**Nominal Value**	
Density/Specific Gravity (23 °C)	1.24 to 1.26 g/cm^3^	1.03 to 1.08 g/cm^3^	1.04 to 1.06 g/cm^3^
Melt Mass-Flow Rate:			
190 °C/2.16 Kg	2.8 to 23 g/10 min	–	–
200 °C/5 Kg	–	–	2.2 to 10 g/10 min
220 °C/10.0 Kg	–	1.0 to 36 g/10 min	–
Molding Shrinkage:			
Flow: 23 °C·(mm/mm)	3.7 × 10^−3^ to 4.1 × 10^−3^	3.9 × 10^−3^ to 6.5 × 10^−3^	4.5 × 10^−3^ to 6.1 × 10^−3^
23 °C	0.30 to 1.1%	0.40 to 0.70%	0.50 to 0.55%
**Mechanical Properties (23 °C)**			
Tensile Modulus	890 to 3647 MPa	1697 to 2826 MPa	1565 to 2290 MPa
Tensile Strength	17.6 to 64 MPa	32 to 53 MPa	20 to 31 MPa
Tensile Strength Yield	15.5 to 72 MPa	29 to 57 MPa	16.4 to 30 MPa
Tensile Strength Break	13.7 to 70 MPa	15 to 50 MPa	16 to 30 MPa
Tensile Elongation Yield	9.8 to 10%	2 to 21%	4 to 61%
Tensile Elongation Break	0.50 to 19%	0.90 to 57%	27 to 63%
Flexural Modulus	2275 to 4495 MPa	1420 to 2770 MPa	1372 to 2454 MPa
Flexural Strength	57.6 to 109 MPa	44.6 to 89 MPa	20.6 to 64.6 MPa
**Thermal Properties**			
Glass Transition Temperature	56.6 to 57.7 °C	100 °C	100 °C
Melting Temperature	190 to 240 °C	210 to 240 °C	230 to 250 °C
**Material Source**	Hatchbox	Hatchbox	Gizmodorks
	www.hatchbox3d.com	www.hatchbox3d.com	www.gizmodorks.com

**Table 2:** Printing parameters.

Parameters, Units	Value
**Infill**	
Pattern	Rectilinear
Angle, °	45°/−45°
Density, %	100
**Speed**	
Travel speed, mm/s	100
Retraction speed, mm/s	5
Infill speed, mm/s	25
**Quality**	
Flow rate, %	100
Layer Thickness, mm	0.3
No. of perimeters	3
**Temperature**	
Bed, °C	90
Nozzle, °C	240

**Table 3:** Dimensions of the printed specimens.

No	Material	Length (L)	Thickness (T)	Width Overall (WO)	Width Narrow (W)
1	ABS	165.12	3.66	19.10	13.20
2	PLA	165.10	3.60	19.08	13.15
3	HIPS	165.00	3.58	19.04	13.43
4	ABS-PLA-ABS	165.20	3.57	19.32	13.50
5	ABS-HIPS-ABS	165.15	3.62	19.13	13.25
6	PLA-ABS-PLA	165.00	3.65	19.30	13.50
7	PLA-HIPS-PLA	165.08	3.60	19.10	13.45
8	HIPS-PLA-HIPS	164.95	3.68	19.19	13.50
9	HIPS-ABS-HIPS	165.13	3.64	19.13	13.35

**Table 4:** Experimental results on the tensile stress-strain response for the homogeneous and sandwich- structured specimens.

No	Material(s)	Tensile Stress (MPa)		Tensile Elongation at Ereak (mm)		Young’s Modulus (MPa)	
		Mean	StdDev	Mean	StdDev	Mean	StdDev
1	ABS	32.89	2.32	4.71	1.03	1049.78	54.81
2	PLA	47.46	2.37	4.16	0.20	1396.90	47.33
3	HIPS	20.06	0.26	6.69	1.92	933.33	14.69
4	ABS-PLA-ABS	38.28	0.16	6.02	0.07	1232.96	0.78
5	ABS-HIPS-ABS	29.67	0.07	5.16	0.52	1049.05	4.87
6	PLA-ABS-PLA	44.40	0.26	6.14	0.09	1364.27	16.68
7	PLA-HIPS-PLA	38.77	1.18	5.38	0.38	1351.27	2.50
8	HIPS-PLA-HIPS	25.87	0.06	8.57	1.56	981.45	141.42
9	HIPS-ABS-HIPS	22.21	0.05	6.10	0.12	992.02	3.59
